# LncRNA OIP5-AS1 Regulates the Warburg Effect Through miR-124-5p/IDH2/HIF-1α Pathway in Cervical Cancer

**DOI:** 10.3389/fcell.2021.655018

**Published:** 2021-08-26

**Authors:** Li Li, Yan Ma, Kamalibaike Maerkeya, Davuti Reyanguly, Lili Han

**Affiliations:** ^1^Department of Gynecology, Affiliated Tumor Hospital of Xinjiang Medical University, Urumqi, China; ^2^Department of Gynecology, People’s Hospital of Xinjiang Uygur Autonomous Region, Urumqi, China

**Keywords:** lncRNA, cervical cancer, Warburg effect, OIP5-AS1, hypoxia

## Abstract

Hypoxia reprogrammed glucose metabolism affects the Warburg effect of tumor cells, but the mechanism is still unclear. Long-chain non-coding RNA (lncRNA) has been found by many studies to be involved in the Warburg effect of tumor cells under hypoxic condition. Herein, we find that lncRNA OIP5-AS1 is up-regulated in cervical cancer tissues and predicts poor 5-years overall survival in cervical cancer patients, and it promotes cell proliferation of cervical cancer cells *in vitro* and *in vivo*. Moreover, OIP5-AS1 is a hypoxia-responsive lncRNA and is essential for hypoxia-enhanced glycolysis which is IDH2 or hypoxia inducible factor-1α (HIF-1α) dependent. In cervical cancer cells, OIP5-AS1 promotes IDH2 expression by inhibiting miR-124-5p, and IDH2 promotes the Warburg effect of cervical under hypoxic condition through regulating HIF-1α expression. In conclusion, hypoxia induced OIP5-AS1 promotes the Warburg effect through miR-124-5p/IDH2/HIF-1α pathway in cervical cancer.

## Introduction

Many life activities, such as growth, reproduction, maintaining their structure, and responding to the external environment, require energy consumption, which are mainly supplied by adenosine triphosphate (ATP), and sugar metabolism is the most important ATP generation pathway. It has been recognized for a long time that cancer cells have a unique way of glucose metabolism, Warburg effect, characterized by even in an oxygen-enriched environment, cancer cells are mainly powered by glycolysis, and oxidative phosphorylation is reduced (Warburg, 1956). The Warburg effect guarantees the advantages of tumor cell growth, not only can provide sufficient energy for high-energy metabolism, but also ensure that less reactive oxygen species (ROS) are produced in the mitochondria (Ferreira, 2010; Liberti and Locasale, 2016). Although the Warburg effect is considered a potential target for curing cancer, the mechanism of the Warburg effect is only been found at the tip of the iceberg (Chen et al., 2007).

There is 75% of human genomic DNA being transcribed into RNA, but of only 2% of the genome encodes proteins, and 98% of transcripts are non-coding RNA (Ulitsky, 2016; Zhao et al., 2016). A single-stranded RNA molecule with a length of about 20–24 nucleotides is called a non-coding single-stranded RNA molecule, and a non-coding RNA with a length of more than 200 nucleotides is called a long-chain non-coding RNA (lncRNA) (Ulitsky, 2016; Zhao et al., 2016). LncRNA was originally considered to be “junk” RNA, but in recent years researches has found that lncRNA plays an important role in many life activities such as dose compensation effect, epigenetic regulation, cell cycle regulation, and cell differentiation regulation (Mercer et al., 2009; Boon et al., 2016). In recent years, with the continuous research on the regulation of energy metabolism of tumor cells by microRNAs (miRNAs) which are also non-coding RNA, it has been suggested that lncRNAs with longer sequences and more complex spatial structures may play a role in the regulation of tumor cell energy metabolism (Redis et al., 2016; Anastasiadou et al., 2018). Many previous studies confirm this hypothesis that lncRNA can regulate key steps in the glucose metabolism (Fan et al., 2017; Sun et al., 2018), lipid metabolism (Chen, 2016; Huang et al., 2017), and amino acid metabolism (Yang et al., 2013; Rion and Rüegg, 2017) pathways of tumor cells, so that tumor cells are in a high metabolic state of glucose, fatty acids, amino acids, etc., and provide the necessary energy and material basis for the survival of tumor cells (Schmitt and Chang, 2016; Cen et al., 2017).

Gene sequencing technology revolutionizes new discoveries in cancer research. Here we report a novel lncRNA obtained by sequencing technology that is abnormally expressed in cervical cancer tissues, OIP5-AS1, is highly expressed in cervical cancer tissues and predicts poor overall survival in cervical cancer patients, and has been reported to promote proliferation and invasion, and inhibit cell apoptosis by sponging miR-143-3p (Chen et al., 2019; Yang et al., 2019; Song et al., 2020). We found that OIP5-AS1 was a hypoxia-responsive lncRNA and was essential for hypoxia-enhanced glycolysis which is IDH2 or hypoxia inducible factor-1α (HIF-1α) dependent. In addition, OIP5-AS1 promotes IDH2 expression by inhibiting miR-124-5p, and IDH2 promotes the Warburg effect of cervical under hypoxic condition through regulating HIF-1α expression. All in all, the results in the present study suggest that hypoxia induced OIP5-AS1 promotes the Warburg effect through miR-124-5p/IDH2/HIF-1α pathway in cervical cancer.

## Materials and Methods

### Patients and Ethics Statement

All cervical cancer patients who provided tissues were informed of the content of this study and signed informed consent. Moreover, all protocols related to human tissue samples were reviewed and monitored by the Ethics Committee in Affiliated Tumor Hospital of Xinjiang Medical University.

Total of 89 cervical cancer patients provided tumor tissues and normal adjacent tissues by surgery from January 2018 to July 2018 in the Affiliated Tumor Hospital of Xinjiang Medical University. Inclusion criteria: (1) Cervical cancer patients without any treatment before surgery; (2) with complete information, such as age, gender, imaging examination, FIGPO stage and so on; (3) Complete 5-year follow-up record; (4) Without any other malignant tumor, or other chronic infectious diseases; Exclusion criteria: (1) Loss of follow-up, or death from other illness or accident; (2) Pregnant women or pregnancy, lactating women, as well as or drug users; and (3) Withdrawal from other types of medical treatments.

### Cell Culture and Transfection

Hela, Caski, Siha, Ect1/E6E7, and MS751 cells were purchased from American type culture collection; Hela cell over-expression IDH2 was established by Cyagen Biosciences^1^, and empty plasmid was transferred into Hela cells as a control. All cells were cultured in DMEM (61870044, Thermo Fisher, United States) which was plus with 10% fetal bovine serum (10437028, Thermo Fisher, United States) at 37°C with 5% CO2. We directly transfected 50 nmol/l of siRNA or miRNA (siRNA or miRNA was designed and synthesized by company Sangon Biotech (China), and the sequences of siRNA or miRNA were showed in Table 1) into 2.5 × 10^6^ gastric cancer cells using Lipofectamine 2000 according to the manufacturer’s protocols. After 72 h of transfection, we performed experiments. For wild type (WT) or mutated (MUT) (The sequence complementary to miR-124-5p was changed to the same sequence as miR-124-5p) versions of the 3′-UTR of OIP5-AS1 and IDH2 were cloned into pisCHECK2 (97157, Addgene, United States), and then began transfection into cells as Si-RNA, and used a Dual-Lucy Assay kit (D00100, Solarbio, China) to detect luciferase activity following the manufacturer’s protocol. After 72 h, gene expression was determined by reverse transcription-quantitative (RT-q) PCR or western blot.

**TABLE 1:** Sequences of primers of qPCR, si-RNA, and miRNA.

Primers used in RT-qPCR analysis	
OIP5-AS1	Forward: 5′-GACGTGCATTCCAACCAACC-3′
	Reverse: 5′-GTGTGGGTGCAAGCAGAAAG-3′
miR-124-5p	Forward: 5′-ACACTCCAGCTGGGCGGTTCACAGCGGAC-3′
	Reverse: 5′-TGGTGTCGTGGAGTCG-3′
LDHA	Forward: 5′-ACCCAGTTTCCACCATGATT -3′
	Reverse: 5′-CCCAAAATGCAAGGAACACT-3′
GLUT1	Forward: 5′-CGGGCCAAGAGTGTGCTAAA-3′
	Reverse: 5′-TGACGATACCGGAGCCAATG-3′
U6	Forward: 5′-CTCGCTTCGGCAGCACA-3′
	Reverse: 5′-AACGCTTCACGAATTTGCGT-3′
β-actin	Forward: 5′-GGCTGTATTCCCCTCCATCG-3′
	Reverse: 5′-CCAGTTGGTAACAATGCCATGT-3′
Si-RNA sequence	
Si-NC	5′-CTAGCTTAAAGCGCCGTTACGT-3′
Si-AS1-1	5′-AUAAACAGGCUUGUUGUUCAC-3′
Si-AS1-2	5′-UGAGAAUGUACUUUGUGAGAU-3′
Si-AS1-3	5′-UACUAAUAAUUUCAUUCUCAG-3′
Si- IDH2	5′-UGAUACAGGAUUAACCUUGAA-3′
miRNA regulate sequence	
miR-124-5p-NC	5′-TTACGCACGGGAACTTCAACGT-3′
miR-124-5p-mimic	5′-CGTGTTCACAGCGGACCTTGAT-3′
miR-124-5p-inhibitor	5′-GCACAAGTGTCGCCTGGAACTA-3′
Primers used in RIP RT-qPCR	
OPI5-AS1 (FL)	Forward: 5′-GCTGATGCCAGGGTCTTGAT-3′
	Reverse: 5′-TGGTGTGTCCGTTGGAACTT-3′
OPI5-AS1 (F1)	Forward: 5′-ACAGCTTCTACGGCGGAAAT-3′
	Reverse: 5′-TTTCGATCCGGATTGGGTCC-3′
OPI5-AS1 (F2)	Forward: 5′-CTGCGAAGATGGCGGAGTAA-3′
	Reverse: 5′-TCAACTGATACCGCTGACGG-3′

### Real-Time Quantitative Polymerase Chain Reaction

Cells and tissues were all used RNAiso plus (9109, TAKARA, Japan) to lysis. At last, phenol chloroform/isopropanol was used to extract total RNA from cells (Poong et al., 2017; Toni et al., 2018). After preparing the cDNA using a PrimeScript RT reagent Kit with gDNA eraser (RR047A, Takara, Japan), 20 μL of qPCR system was prepared and analyzed as the describe in the instructions of GoTaq qPCR Master Mix (A6001, promega, United States). The relative expression of gene was calculated by 2^–ΔΔCt^ method, and β-actin or U6 was used as a loading control. Primers was showed in Table 1.

### Cellular Immunofluorescence

Cells were fixed with 4% paraformaldehyde for 15 min at room temperature. After being blocked with 5% of BSA in 0.3 Triton X-100 for 1 h at room temperature, antibody was added to incubate the cell. At last, all slides were counterstained the nucleus with 5 μg/mL DAPI for 5 min at room temperature.

### FISH Analysis

Fluorescence *in situ* hybridization (FISH) was performed as previously described (Chen Z.-Z. et al., 2016). In brief, after fixing and blocking, cells or tissues were incubated with a fluorescent probe for binding to the human version of the OIP5-AS1 gene which was synthesized by Genomeditech Co., Ltd. For cells, it should be counterstained the nucleus with 5 μg/mL DAPI for 5 min at room temperature. At last, all samples were analyzed by confocal microscopy.

### Cell Viability Assay

2 × 10^3^ cells were seeded into 96-well cell culture plate and normal cultured for 72 h. And we measured the cell viability using a Cell Proliferation Assay Kit (C0009, Beyotime Scientific, China) (Jiang et al., 2014; Liu et al., 2015).

### Cell Clone Test

About 0.8 × 10^6^ cells were seeded into in six-well plates. We changed the medium every 3 days and stopped the culture when we saw visible clones, and counted the cell clone after staining with 0.25% crystal violet for 25 min.

### Xenograft Mouse Model

5 × 10^6^/0.2 ml Hela cells were injected into the six-weeks old nude mouse in middle axillary lateral skin (*n* = 7 each group). Mice were fed properly 10–14 days after cell inoculation. Mice were sacrificed, and tumor tissues were taken out and recorded the weight of tumor.

### Western Blot Analysis

Levels of protein expression was analyzed by western blot analysis as previously described (Zhao et al., 2015; Tao et al., 2018). Briefly, total of 50 μg total protein in cell lysate was separated by a 10% SDS-PAGE under a 90 V constant voltage. Then we transferred the protein from the SDS-PAGE gel to the PVDF membrane. Antibody information is displayed on Table 2. At last, a Bio-Rad Imaging system was used to detect immunoreactivity, and Image J (V2.1.4.7, NIH, United States) was used to quantify the gray value of protein bands.

**TABLE 2:** Antibody information.

Antibody	Dilution ratio	Cat. no.	Manufacturer
IDH2	1:1000	56439	Cell Signaling Technology
LDHA	1:1500	3582	Cell Signaling Technology
GLUT1	1:500	ab115730	ABCAM
HIF-1α	1:1000	ab1	ABCAM
β-actin	1:5000	HC201-02	TransGen Biotech
Goat anti-Rabbit IgG (H + L)-Alexa Fluor 488	1:500	A11008	Invitrogen
Goat Anti-Rabbit IgG H&L (HRP)	1:2000	ab97051	ABCAM
Goat Anti-mouse IgG H&L (HRP)	1:2000	Ab205719	ABCAM

### Lactate Production and Glucose Uptake Assay

Human cervical cancer cells were cultured in glucose-free DMEM for 16 h, and then replaced the culture incubated with high-glucose DMEM under normoxic or hypoxic conditions for an additional 24 h. At last, we harvested cells to detect the intracellular glucose levels using a PicoProbe^TM^ Glucose Fluorometric Assay Kit (K688, BioVision, United States) as the describe in manufacturer’s instructions, and harvested the culture medium to detect lactate levels in the culture medium using a PicoProbe^TM^ Lactate Fluorometric Assay Kit (K688, BioVision, United States) as the describe in manufacturer’s instructions.

### Intracellular α-KG, 2-HG, ROS, and NADP+/NADPH Assay

The human cervical cancer cells were collected, and we used a Alpha-Ketoglutarate Colorimetric/Fluorometric Assay Kit (K677, BioVision, United States) to determine intracellular levels of α-KG, and used a PicoProbe^TM^ D-2-Hydroxyglutarate Dehydrogenase Assay Kit to determine intracellular levels of 2-HG (K248, BioVision, United States), used a NADP/NADPH Quantitation Colorimetric Kit (K347, BioVision, United States) to detect NADP/NADPH with total protein as a normalized standard. Intracellular levels of ROS were measured by flow cytometry.

### Carbon-Labeled Isotopologues Analysis

Cells were seeded in six-well cell culture plate and we used C13-labeled glucose and C13-labeled glutamine cell culture medium to replace normal cell culture medium. After 24 h, we harvest the cell and lysed cells with lysate buffer (20 mmol/L Tris–HCl, 2.5 mmol/L EDTA, 150 mmol/L NaCl, 0.5% NP-40, pH = 8.0). At last, cell lysate was analyzed by a Gas chromatography–mass spectrometry.

### Statistical Analysis

SPSS 20.0 (IBM, United States) is used to analyze the data in the present study. *T* test and chi-square test are used to compare the difference between two groups, and one-way ANOVA with duncan test as *post hoc* test to compare the difference between multiple groups. Log-rank (Mantel-Cox) test is used to compare the survival of patients with high and low OIP5-AS1 expression. *P* < 0.05 indicates significant difference.

## Results

### OIP5-AS1 Is Up-Regulated in Cervical Cancer

**FIGURE 1:**
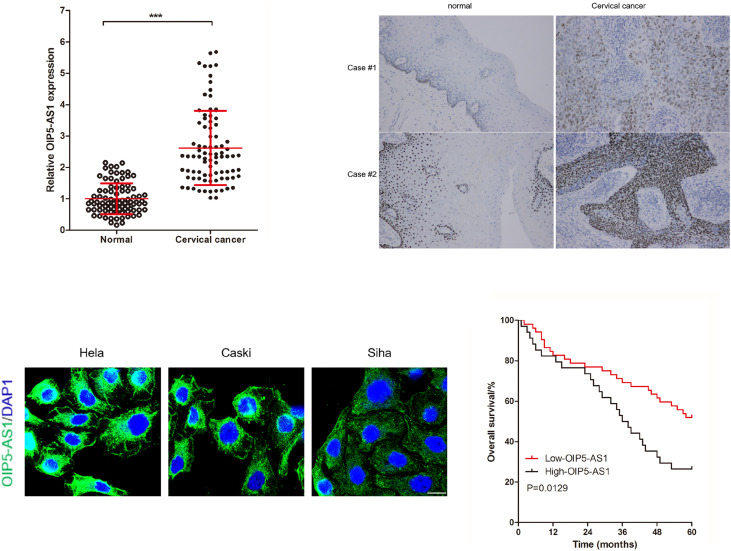
OIP5-AS1 is up-regulated in cervical cancer tissues and related to poor prognosis in patients with cervical cancer. **(A)** RT-qPCR analysis of the indicated OIP5-AS1 levels in 86 pairs of cervical cancer tissues and matched adjacent normal cervical tissues. Data are shown as (mean ± SD), *** was *P* < 0.001 and was calculated by paired *t* test. **(B,C)** OIP5-AS1 expression in tissues **(B)** and Hela, Caski, and Siha cells **(C)** were analyzed by FISH staining. **(D)** High expression of OIP5-AS1 shows poor prognosis in patients with cervical cancer. *P* value was calculated by Log-rank (Mantel-Cox) test.

To analyze the expression of OIP5-AS1 in cervical cancer tissues, we collected 86 pairs of cervical cancer tissues and matched adjacent normal cervical tissues, and detected OIP5-AS1 using RT-qPCR analysis. As the results of previous research (Yang et al., 2019), the results showed that the expression of OIP5-AS1 in cervical cancer tissues were significantly higher than that in matched adjacent normal cervical tissues (Figure 1A). Additionally, we also used FISH staining to measure OIP5-AS1 in tissues (Figure 1B) and cervical cancer cells (Hela, Caski, and Siha), and found that OIP5-AS1 was mainly cytoplasmic localization in cervical cancer cells (Figure 1C). According to the expression of OIP5-AS1 in 86 cases of cervical cancer tissues, we divided these 86 cervical cancer patients into two groups using use the average value of OIP5-AS1 expression as the dividing line, Low-OIP5-AS1 group (OIP5-AS1 expression < the average of OIP5-AS1 expression in 86 cervical cancer patients) and High-OIP5-AS1 group (OIP5-AS1 expression ≥ the average of OIP5-AS1 expression in 86 cervical cancer patients). To analyze the correlation between OIP5-AS1 expression and clinical features in cervical cancer patients, and found that (Table 3) OIP5-AS1 expression in cervical cancer tissues was significantly related to tumor size, differentiation, lymph node metastasis and FIGO stages of cervical cancer. In addition, we also found that high levels of OIP5-AS1 correlated with poor 5-years overall survival (Figure 1D).

**TABLE 3:** Relationship between lncRNA OIP5-AS1 expression and clinical data of cervical cancer patients (*n*).

Subjects	OIP5-AS1		χ^2^	*P*
	
	High expression (*n* = 34)	Low expression (*n* = 52)		
Age (year)				
≥45	13	23	0.304	0.582
<45	21	29		
Histomorphology				
Squamous cell carcinoma	15	20	0.379	0.827
Adenocarcinoma	16	28		
Adenosquamous carcinoma	3	4		
Tumor size (cm)				
>4	23	14	13.909	<0.0001
≤4	11	38		
Differentiation				
Low	16	15	8.272	0.016
Medium	12	12		
High	6	25		
Lymph node metastasis				
Yes	3	8	4.460	0.035
No	31	44		
Distal metastasis				
Yes	1	4	0.847	0.357
No	33	48		
FIGO stages				
I	18	12	14.442	0.003
II	13	26		
III–IV	2	15		
HPV				
HPV16+	16	17	1.875	0.392
HPV18+	15	28		
Others	3	7		

### OIP5-AS1 Promotes Cell Proliferation in Cervical Cancer

**FIGURE 2:**
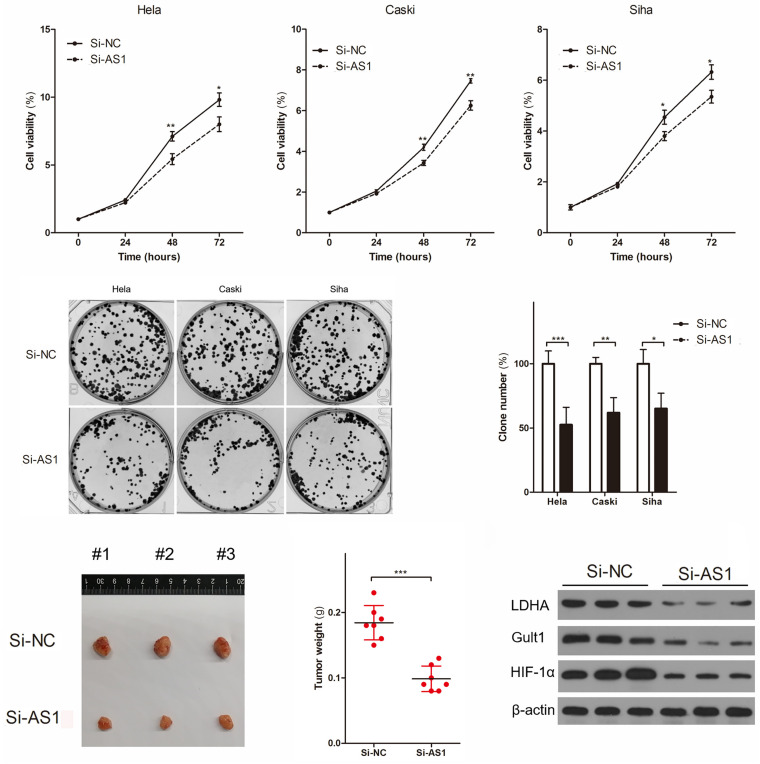
OIP5-AS1 regulates the proliferation of cervical cancer cell *in vivo* and *in vitro*. **(A–C)** Cell viability **(A)** and cell clone number **(B,C)** were detected in Hela, Caski, and Siha cervical cancer cell knocking down OIP5-AS1. Data were shown as mean ± SD of three individual experiments. * was *P* < 0.05, ** was *P* < 0.01, and *** was *P* < 0.001, and *P* value was calculated by Student’s *t* test. **(D,E)** Representative of Hela cervical cancer cell xenograft tumors images are showed **(D)** and the xenograft tumors weight was compared **(E)**. Data were shown as mean ± SD. *** was *P* < 0.001, and *P* value was calculated by Student’s *t* test. **(F)** Western blot analysis shows LDHA, GLUT1 and HIF-1α protein in Hela cervical cancer cell xenograft tumors knocking down OIP5-AS1 and its controls.

To investigate the functional of OIP5-AS1, we establishedOIP5-AS1 knock-down cervical cancer cell lines by transfection siRNA of OIP5-AS1 (Si-AS1), and being successfully verified by RT-qPCR analysis (Supplementary Figure 1A) and FISH staining (Supplementary Figure 1B). RT-qPCR analysis showed that (Supplementary Figure 1A) Si-AS1-1 could decrease OIP5-AS1 (up to 20%) in cervical cancer cells. Therefore, if there is no special instruction, we will use Si-AS1-1 to knock down the expression of OIP5-AS1 in the following research. As the results of previous research (Yang et al., 2019), the results of cell proliferation test *in vitro* and *in vivo* showed that OIP5-AS1 knocked down by Si-AS1 was significantly decreased cell viability (Figure 2A), number of cell clone (Figures 2B,C) and the xenograft tumors weight (Figures 2D,E) in cervical cancer. Furthermore, we performed western blot analysis of protein expression related to energy metabolism in the xenograft tumors of Hela, and found that OIP5-AS1 knocked down by Si-AS1 could decreased the expression of LDHA, GULT1, and HIF-1α protein in the xenograft tumors of Hela (Figure 2F), suggesting that OIP5-AS1 might be a oncogene which was related to energy metabolism in cervical cancer.

### OIP5-AS1 Promotes Hypoxia-Enhanced Warburg Effect

**FIGURE 3:**
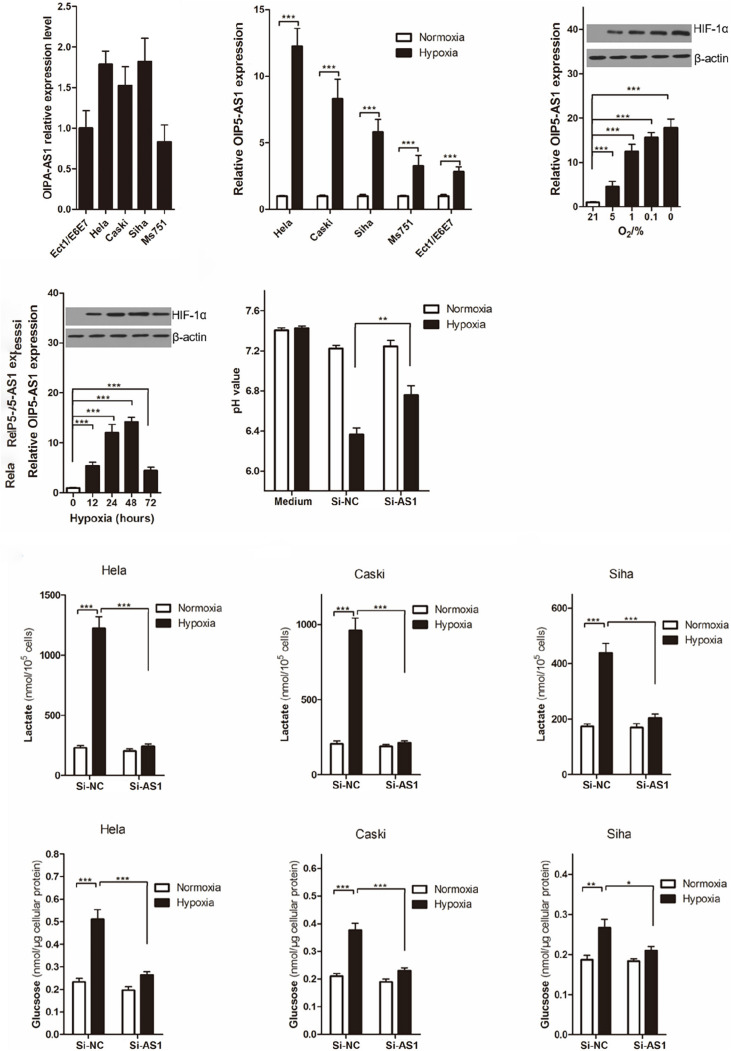
OIP5-AS1 mediates the promoting effect of hypoxia on Warburg effect in cervical cancer cell. **(A)** RT-qPCR analysis was used to quantify the copy numbers of OIP5-AS1 transcript per cell in the indicated tumor cells and non-transformed cells. Data were shown as mean ± SD of three individual experiments. **(B)** We cultured Hela, Caski, and Siha cells under 20% O_2_ (normoxic) or 1% O_2_ (hypoxic) for 24 h, and RT-qPCR analysis of the indicated OIP5-AS1 levels. Data are shown as (mean ± SD), *** was *P* < 0.001 and *P* value was calculated by Student’s *t* test. **(C,D)** RT-qPCR analysis of the indicated OIP5-AS1 levels in Hela cells under different oxygen concentrations **(C)** or under hypoxia for 24 h **(D)**. Western blot analysis shows HIF-1α protein in Hela cervical cancer cell. Data are shown as (mean ± SD), *** was *P* < 0.001 and *P* value was calculated by *post hoc* comparisons. **(E)** The pH value of Hela cells knocking down OIP5-AS1 or its control were detected. Data are shown as (mean ± SD), ** was *P* < 0.01 and *P* value was calculated by Student’s *t* test. **(F,G)** Levels of lactate **(F)** in the culture medium and intracellular glucose **(G)** in the culture medium of Hela, Caski, and Siha cells after culturing for 24 h under hypoxia. Data were shown as mean ± SD of three individual experiments. * was *P* < 0.05, ** was *P* < 0.01 and *** was *P* < 0.001, and *P* value was calculated by Student’s *t* test.

Dependence on glycolytic energy supply is one of the characteristics of tumor cells, and as a first attempt to investigate whether OIP5-AS1 regulate the Warburg effect under hypoxic conditions, we first quantified the amount of OIP5-AS1 in cervical cancer cells (Hela, Caski, Siha, Ms751) and immortalized human cervical squamous cells (Ect/E6E7) using RT-qPCR analysis. As the results of previous research (Yang et al., 2019), levels of OIP5-AS1 in cervical cancer cells (Hela, Caski, Siha, Ms751) were significantly higher than that in immortalized human cervical squamous cells (Ect/E6E7) (Figure 3A). Then we compared the expression change of OIP5-AS1 between cultured in normoxia condition (20% O_2_ and 24 h) and hypoxia condition (1% O_2_ and 24 h), and found that levels of OIP5-AS1 expression was significantly increase in hypoxia condition (Figure 3B). Moreover, levels of OIP5-AS1 also been found induced by hypoxia in a concentration-dependent (Figure 3C) and time-dependent manner (Figure 3D), and western blot analysis showed that HIF-α expression was also related to hypoxia in a concentration-dependent (Figure 3C) and time-dependent manner. Interestingly, hypoxia induced the pH value of Hela cell culture media significantly reduced, but OIP5-AS1 knockdown by Si-AS1 could significantly this change (Figure 3E). Importantly, hypoxia also induced more lactate production (Figure 3F) and glucose uptake (Figure 3G) in cervical cancer cells (Hela, Caski, and Siha). However, all these hypoxia-induced events about lactate production and glucose uptake were significantly reversed in cervical cancer cells knocking down OIP5-AS1, indicating that OIP5-AS1 promotes hypoxia-enhanced Warburg effect in cervical cancer.

### OIP5-AS1 Regulates TCA Cycle and Redox State

**FIGURE 4:**
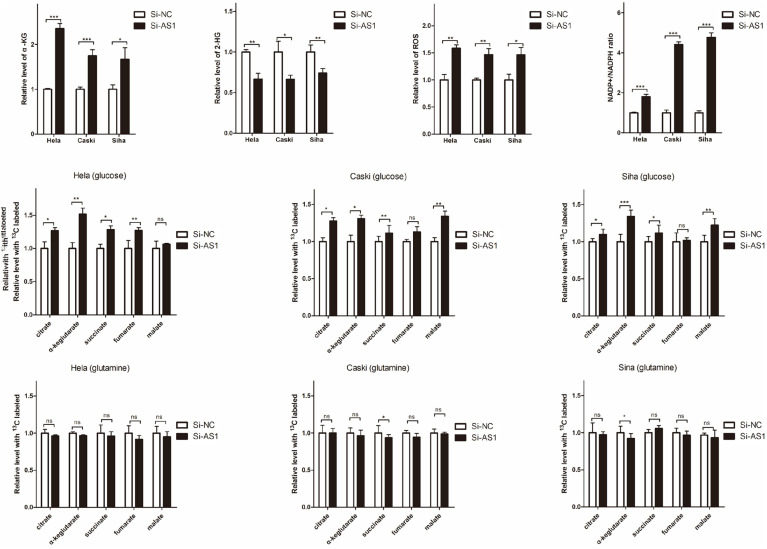
OIP5-AS1 regulates intracellular concentrations of α-KG, 2-HG and redox status in cervical cancer cell. **(A–D)** Levels of intracellular α-KG **(A)**, 2-HG **(B)**, ROS **(C)**, and NADP+/NADPH **(D)** in Hela, Caski, and Siha cells knocking down OIP5-AS1 and its control. Data were shown as mean ± SD of three individual experiments. * was *P* < 0.05, ** was *P* < 0.01, and *** was *P* < 0.001, and *P* value was calculated by Student’s *t* test. **(E,F)** Levels of tricarboxylic acid cycle metabolites were determined in Hela, Caski, and Siha cells knocking down OIP5-AS1 and its control using C13 marked glucose **(E)** or glutamine **(F)**. Data were shown as mean ± SD of three individual experiments. ns was *P* > 0.05, * was *P* < 0.05 and ** was *P* < 0.01, and *P* value was calculated by Student’s *t* test.

To investigate the effects of OIP5-AS1 expression in the form of cervical cancer energy supply, we analyzed the changes of intracellular α-KG, 2-HG, ROS, and ratio of NADP+/NADPH in cervical cancer cells. Compared with cervical cancer cells transfection to Si-NC, intracellular α-KG levels of cervical cancer cells knocking down OIP5-AS1 by transfection to Si-AS1 was significantly increase (Figure 4A), but levels of intracellular 2-HG was significantly decrease (Figure 4B), and the levels of ROS (Figure 4C) and ratio of NADP+/NADPH (Figure 4D) were all significantly increase. Additionally, to identify the metabolic flux that contributes to changes, carbon-labeled glucose or glutamine was added in the culture medium and TCA cycle metabolites were measured, and found that (Figure 4E) OIP5-AS1 increased all metabolites in TCA cycle from C13-glucose, slightly decreased all metabolites in TCA cycle from C13-glutamine excepting significantly increased in α-KG, suggesting that OIP5-AS1 affects cervical cancer energy metabolism may be related to TCA cycle, especially α-KG metabolism.

### OIP5-AS1 Promotes Hypoxia-Enhanced Warburg Effect Is IDH2 Dependent

**FIGURE 5:**
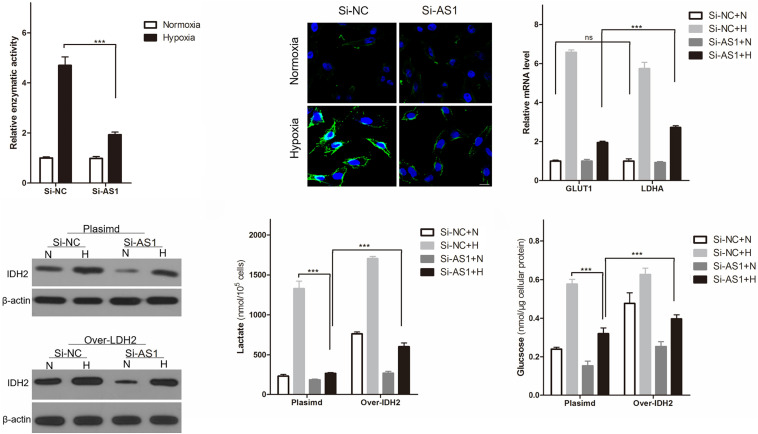
OIP5-AS1 mediates the promoting effect of hypoxia on Warburg effect is IDH2 dependent. **(A)** Levels of LDHA enzymatic activity **(A)**, representative of GLUT1 protein expression images **(B)**, and levels of GLUT1 and LDHA mRNA **(C)** in Hela cells knocking down OIP5-AS1 or its control under normoxic or hypoxic conditions for 24 h. Data were shown as mean ± SD of three individual experiments. ns was *P* > 0.05, *** was *P* < 0.001, and *P* value was calculated by Student’s *t* test. **(D)** We established a Hela cells overexpressing IDH2 (Over-IDH2) and its control cells (Plasmid), and used western blot to detect the expression of IDH2 protein in Over-IDH2 or Plasmid Hela cells after knocking down OIP5-AS1. **(E,F)** Levels of lactate **(E)** in the culture medium and intracellular glucose **(F)** of Hela cells after culturing for 24 h under hypoxia. Data were shown as mean ± SD of three individual experiments. *** was *P* < 0.001 and *P* value was calculated by *post hoc* comparisons.

Consistent with the above findings that OIP5-AS1 promoted glucose uptake and lactate production, hypoxia induced an increase in LDHA enzymatic activity, GLUT1 and LDHA expression levels in Hela cells, and knockdown of OIP5-AS1 could significantly reverse it (Figures 5A–C). Base on the above finding, OIP5-AS1 affects cervical cancer energy metabolism may be related to TCA cycle, especially α-KG metabolism (Figure 4). And hypoxia induced IDH2 consumes α-KG by carboxylating α-KG to citrate (Terunuma et al., 2014), and could induce production of 2-HG by LDHA and MDH1/2 (Intlekofer et al., 2015). To explore doesOIP5-AS1 affect Warburg effect through IDH2/α-KG-mediated metabolic changes? We first established a Hela cell line overexpressing IDH2, and western blot analysis (Supplementary Figure 2A) and cellular immunofluorescence (Supplementary Figure 2B) confirmed that Hela cell line overexpressing IDH2 (Over-IDH2) was successfully constructed. Western blot analysis showed that hypoxia induced increased IDH2 expression, but knock down of OIP5-AS1 could significantly reverse hypoxia-induced elevation of IDH2 expression (Figure 5D). The similar change also incident also appeared in Hela cell line overexpressing IDH2 (Figure 5D). Importantly, overexpression of IDH2 can indeed be reversed reduced lactate production (Figure 5E) and reduced glucose intake (Figure 5F) caused by OIP5-AS1 knockdown, showing OIP5-AS1 promotes hypoxia-enhanced Warburg effect is IDH2 dependent.

### OIP5-AS1 Promotes IDH2 by Inhibiting miR-124-5p

**FIGURE 6:**
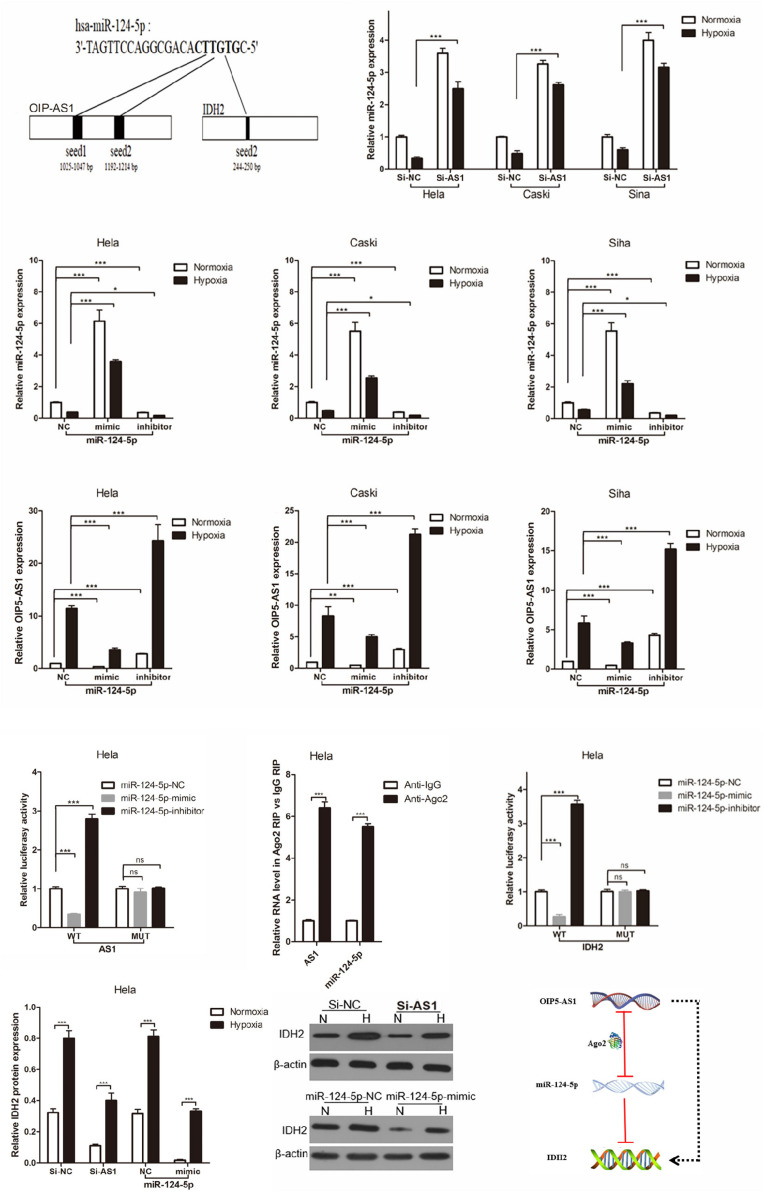
OIP5-AS1 regulates miR-124-5p expression by both directly targeting and Ago2-dependent manner, and miR-124-5p target inhibition of IDH2 expression in cervical cancer cell. **(A)** Predicted binding sites of miR-124-5p on OIP5-AS1 transcript (black) and IDH2 transcript (black). **(B)** RT-qPCR analysis of the indicated miR-124-5p levels in Hela, Caski, and Siha cells under normoxic or hypoxic conditions for 24 h. Data were shown as mean ± SD of three individual experiments. *** was *P* < 0.001 and *P* value was calculated by Student’s *t* test. **(C,D)** RT-qPCR analysis of the indicated miR-124-5p or OIP5-AS1 levels in Hela, Caski, and Siha cells over-expressing miR-124-5p or expressing knockdown miR-124-5p under normoxic or hypoxic conditions for 24 h. Data were shown as mean ± SD of three individual experiments. * was *P* < 0.05, ** was *P* < 0.01 and, *** was *P* < 0.001, and *P* value was calculated by Student’s *t* test. **(E)** miR-124-5p-NC, mimic and inhibitor was co-transferred with OIP5-AS1 wild type (WT) or mutant (MUT) transcript, and then detected luciferase activity. Data were shown as mean ± SD of three individual experiments. ns was *P* > 0.05 and *** was *P* < 0.001, and *P* value was calculated by *post hoc* comparisons. **(F)** RT-qPCR analysis of the indicated miR-124-5p and OIP5-AS1 levels associated with AGO2 after RIP assay in Hela cells. Data were shown as mean ± SD of three individual experiments. *** was *P* < 0.001, and *P* value was calculated by *post hoc* comparisons. **(G,H)** Dual-luciferase reporter assay shows that miR-124-5p targets inhibition of IDH2 expression **(G)**, and Western blot analysis shows that OIP5-AS1 promotes IDH2 expression via inhibiting miR-124-5p expression **(H)**. Data were shown as mean ± SD of three individual experiments. ns was *P* > 0.05 and *** was *P* < 0.001, and *P* value was calculated by *post hoc* comparisons in panel **(G)** and by Student’s *t* test in panel **(H)**. **(I)** Schematic illustration of the proposed model depicting OIP5-AS1 promotes IDH2 expression via inhibiting miR-124-5p expression.

To investigate the molecular mechanism by which OIP5-AS1 regulates miR-124-5p, we used public databases (UCSC, miRBase, and BiBiserv2) to find clues. Interestingly, we found that miR-124-5p as a bridge connecting OIP5-AS1 and IDH2, not only because miR-124-5p was a Warburg effect-related miRNA, but also miR-124-5p had same binding sites to OIP5-AS1 and IDH2 (Figure 6A). Although hypoxia induced miR-124-5p expression to decrease in cervical cancer cells, knocking down OIP5-AS1 could significantly increase miR-124-5p expression in normoxic and hypoxic condition (Figure 6B). And then we regulated miR-124-5p expression by transfection miR-124-5p-NC, -mimic and inhibitor into cervical cancer cells (Figure 6C). Importantly, over-expression of miR-124-5p by transfection miR-124-5pmimic could significantly reduce OIP5-AS1 expression, and knocking down of miR-124-5p by transfection miR-124-5p-inhibitor could significantly increase OIP5-AS1 expression in normoxic and hypoxic condition (Figure 6D). Consistent with the above findings that miR-124-5p inhibited OIP5-AS1 expression in cervical cancer cells (Hela, Caski, and Siha), dual fluorescent gene reporting system shows the luciferase activity of OIP5-AS1-wt was significantly decreased by miR-124-5p mimics, while miR-124-5p had no influence on OIP5-AS1-Mut in Hela cells, suggesting miR-124-5p directly inhibited OIP5-AS1 expression (Figure 6E). Furthermore, an Ago2-dependent manner was important way to regulate target gene by miRNAs. Therefore, we used an anti-Ago2 antibody to analysis the relationship between OIP5-AS1 and Ago2 using RIP assay, and found that OIP5-AS1 and miR-124-5p immunoprecipitated with Ago2 antibody were enhanced relative to IgG control (Figure 6F). Similarly, dual fluorescent gene reporting system and western blot analysis shows miR-124-5p targeted inhibition of IDH2 expression in cervical cancer cells in normoxic and hypoxic condition (Figures 6G,H). Taken together, OIP5-AS1 promotes IDH2 by inhibiting miR-124-5p in cervical cancer cells (Figure 6I).

### OIP5-AS1 Promotes IDH2-Mediated Warburg Effect Is HIF-1α Dependent

**FIGURE 7:**
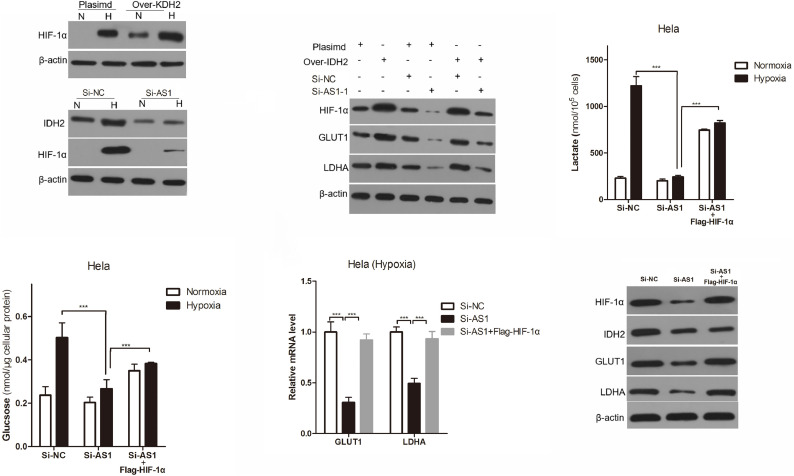
OIP5-AS1 regulates the Warburg effect through IDH2 is HIF-1α dependent. **(A,B)** Levels of IDH2, HIF-1α, GLUT1, and LDHA protein expression in Hela cells using western blot. **(C–F)** Levels of lactate **(C)** in the culture medium and intracellular glucose **(D)** of Hela cells after culturing for 24 h under hypoxia, and RT-qPCR indicated GLUT1/LDHA mRNA levels, western blot analysis showed IDH2, HIF-1α, GLUT1, and LDHA protein expression. Data were shown as mean ± SD of three individual experiments. *P* value was calculated by *post hoc* comparisons. ***P < 0.001.

Hypoxia inducible factor-1α is a key gene for mammalian cells to adapt to hypoxia (Chandel et al., 2000; Zhang et al., 2007) and tumor cell glycolysis dependence (Zhong et al., 1999; Luo et al., 2011), and IDH2 has been found to promote Warburg effect through HIF-1α (Li J. et al., 2018). To explore whether OIP5-AS1 promoted IDH2-mediated Warburg effect through HIF-1α, we first analyzed the expression of HIF-1α after regulating IDH2 expression. As showed in Figure 7A, over-expressing of IDH2 could greatly increase HIF-1α expression, and knocking down of IDH2 expression could greatly decrease the expression of HIF-1α in Hela cells. Additionally, over-expressing of IDH2 could increase the expression of HIF-1α, GLUT1 and LDHA protein expression in Hela under hypoxic environment, while knockdown of OIP5-AS1 decreased the expression of HIF-1α, GLUT1 and LDHA protein expression in Hela under hypoxic condition (Figure 7B). Importantly, knockdown of OIP5-AS1 could reverse elevated HIF-1α, GLUT1 and LDHA protein expression in Hela under hypoxic condition (Figure 7B).

To investigate whether OIP5-AS1 regulates hypoxia enhanced glycolysis through HIF-1α, exogenous HIF-1α was introduced into OIP5-AS1 knockdown cells. Exogenous HIF-1α could not only increase lactate production (Figure 7C) and glucose intake (Figure 7D) in OIP5-AS1 knockdown Hela cells, but also increased the expression of GLUT1 and LDHA expression (Figures 7E,F), indicating that OIP5-AS1 promotes IDH2-mediated Warburg effect is HIF-1α dependent.

## Discussion

According to data released by China National Cancer Center (Chen W. et al., 2016; Feng et al., 2019), the morbidity and mortality of cervical cancer ranks sixth among all malignancies in female, and 131500 new cases and 53000 deaths each year. Although cervical cytology screening has enabled early detection and treatment of cervical cancer and precancerous lesions to greatly reduce the incidence and mortality of cervical cancer, cervical cancer is still one of the important “killers” of women’s health (Jemal et al., 2007; Small et al., 2017). An increasing number of studies have shown that numerous lncRNAs are deregulated in cervical, and previously studies has found that lncRNA plays an important role in cervical cancer pathogenesis and progression through directly regulating target gene or indirectly regulating protein expression via binding to miRNA (Zhang et al., 2016; Wen et al., 2017). In recently years, studies have found that lncRNA is involved in the regulation of tumor metabolism, such as lincRNA-p21 is an important player in the regulation of the Warburg effect in cancer cells through attenuating VHL-mediated HIF-1a ubiquitination and causing HIF-1a accumulation (Yang et al., 2014). LncRNA UCA1, which is highly expressed in bladder cancer tissues, regulates the expression of hexokinase 2 through the mammalian rapamycin protein number and the transcriptional activation factor 3/miR143 signaling pathway, and ultimately promotes the consumption of glucose and lactic acid in bladder cancer cells (Yang et al., 2013). Therefore, further studies on the expression and biological functions of lncRNAs in cervical cancer may facilitate the development of novel therapeutic techniques for patients carrying this malignant tumor.

In this study, we found that the high expression of OIP5-AS1 in cervical cancer tissues was not only related to the poor prognosis of patients with cervical cancer, but also promoted the proliferation of cervical cancer cells *in vitro* and *in vivo*. These results suggest that OIP5-AS1 is an oncogene in cervical cancer, which is similar to Chen et al. (2019); Yang et al. (2019), and Song et al. (2020). Previous study has found that OIP5-AS1 regulates HIF-1α expression in diabetic nephropathy via miR-34a-5p/Sirt1 axis (Li A. et al., 2018). Although this study is not related to tumors, not even in human cells, this study at least suggests that OIP5-AS1 may regulate HIF1-expression. HIF-1α is ubiquitous in human and mammalian cells and is also expressed under normal oxygen (21% O2) (Cheng et al., 2019; Miska et al., 2019), but the synthetic HIF-1 protein is quickly degraded by the intracellular oxygen-dependent ubiquitin protease degradation pathway (Oh et al., 2016; He et al., 2017), and can be stably expressed under hypoxia (Wang and Semenza, 1995; Salceda and Caro, 1997). It was well know that hypoxia contributes to the Warburg effect through the regulation of expression of HIF-1α (Yang et al., 2014; Liu et al., 2016), whether OIP5-AS1 regulates the Warburg effect in cervical cancer cells through HIF-1α remains uncharacterized.

Meaningfully, in the present study, we found that hypoxia induced high expression of OIP5-AS1 in a time- and oxygen-dependent manner in cervical cancer cells, and knockdown of OIP5-AS1 could decrease elevated lactate production and glucose uptake which was induced by hypoxia. Furthermore, OIP5-AS1 also effects intracellular α-KG, 2-HG, ROS, and ratio of NADP+/NADPH in cervical cancer cells. Taken together, suggesting that OIP5-AS1 regulates Warburg effect in cervical cancer. It is well known that lncRNA is non-coding RNAs, they can only exert biological functions by regulating the expression of other genes. To investigate the mechanism of OIP5-AS1 in the regulation of Warburg effect in cervical cancer, we analyzed the sequence in online database, and found that miR-124-5p as a bridge connecting OIP5-AS1 and IDH2. MiR-124-5p has been found to regulate Warburg effect in colorectal cancer DDX6/c-Myc/PTB1 (Sun et al., 2012; Taniguchi et al., 2015). At the same time, miR-124-5p was also been found to regulate lactate transportation in the muscle of largemouth bass (micropterus salmoides) under hypoxia by targeting MCT1 (Zhao et al., 2020). Importantly, in this study, we found that OIP5-AS1 promoted IDH2 expression through suppressing miR-124-5p expression. IDH2, a Isocitrate dehydrogenase, is a key protein in mitochondrial tricarboxylic acid cycle (O’Neill, 2015; Bergaggio et al., 2019). In the regulation of tricarboxylic acid cycle, hypoxia induced IDH2 consumes α-KG by carboxylating α-KG to citrate (Terunuma et al., 2014), and could induce production of 2-HG by LDHA and MDH1/2 (Intlekofer et al., 2015). For Warburg effect, previously study also found that IDH2 promoted the Warburg effect and lung cancer cell growth, which is mediated through HIF1α activation followed by decreased α-KG (Li J. et al., 2018). Therefore, the current results suggest that OIP5-AS1 may be regulate Warburg effect through IDF2-mediated HIF-1α pathway by inhibiting miR-124-5p expression in cervical cancer.

Fortunately, the current study shows that overexpression of IDH2 can indeed be reversed reduced lactate production and reduced glucose intake (Figure 5F) caused by OIP5-AS1 knockdown, showing OIP5-AS1 promotes hypoxia-enhanced Warburg effect is IDH2 dependent. And exogenous HIF-1α could not only increase lactate production and glucose intake in OIP5-AS1 knockdown Hela cells. Taken together, the present study indicates that the high expression of OIP5-AS1 promoted the Warburg effect through miR-124-5p/IDH2/HIF-1α pathway in cervical cancer, and is a potential cervical cancer treatment target.

## Data Availability Statement

The raw data supporting the conclusions of this article will be made available by the authors, without undue reservation.

## Ethics Statement

The studies involving human participants were reviewed and approved by Ethics Committee in Affiliated Tumor Hospital of Xinjiang Medical University. The patients/participants provided their written informed consent to participate in this study. The animal study was reviewed and approved by Ethics Committee in Affiliated Tumor Hospital of Xinjiang Medical University.

## Author Contributions

LL and LH is the guarantor of this work and, as such, had full access to all of the data in the study and took responsibility for the integrity of the data and the accuracy of the data analysis. LH designed the study, drafted the manuscript, and revised the manuscript. LL and YM performed the key experiments. KM and DR participated in the planning of the work and the interpretation of the results. All authors contributed to the article and approved the submitted version.

## Conflict of Interest

The authors declare that the research was conducted in the absence of any commercial or financial relationships that could be construed as a potential conflict of interest.

## Publisher’s Note

All claims expressed in this article are solely those of the authors and do not necessarily represent those of their affiliated organizations, or those of the publisher, the editors and the reviewers. Any product that may be evaluated in this article, or claim that may be made by its manufacturer, is not guaranteed or endorsed by the publisher.

## Footnotes

1
www.cyagen.com

## Supplementary Material

Supplementary Figure 1 Validation of Hela, Caski, and Siha cell line knocking down OIP5-AS1 using RT-qPCR **(A)** and RT-qPCR **(B)**.

Supplementary Figure 2 Validation of Hela cell line overexpressing IDH2 using western blot **(A)** and cellular immunofluorescence **(B)**.
