# Supplementary figures and images for: LncRNA OIP5-AS1 Regulates the Warburg Effect Through miR-124-5p/IDH2/HIF-1α Pathway in Cervical Cancer
Source: Front Cell Dev Biol. 2021 Aug 26;9:655018. doi: 10.3389/fcell.2021.655018 (PMC8427313; doi:10.3389/fcell.2021.655018)

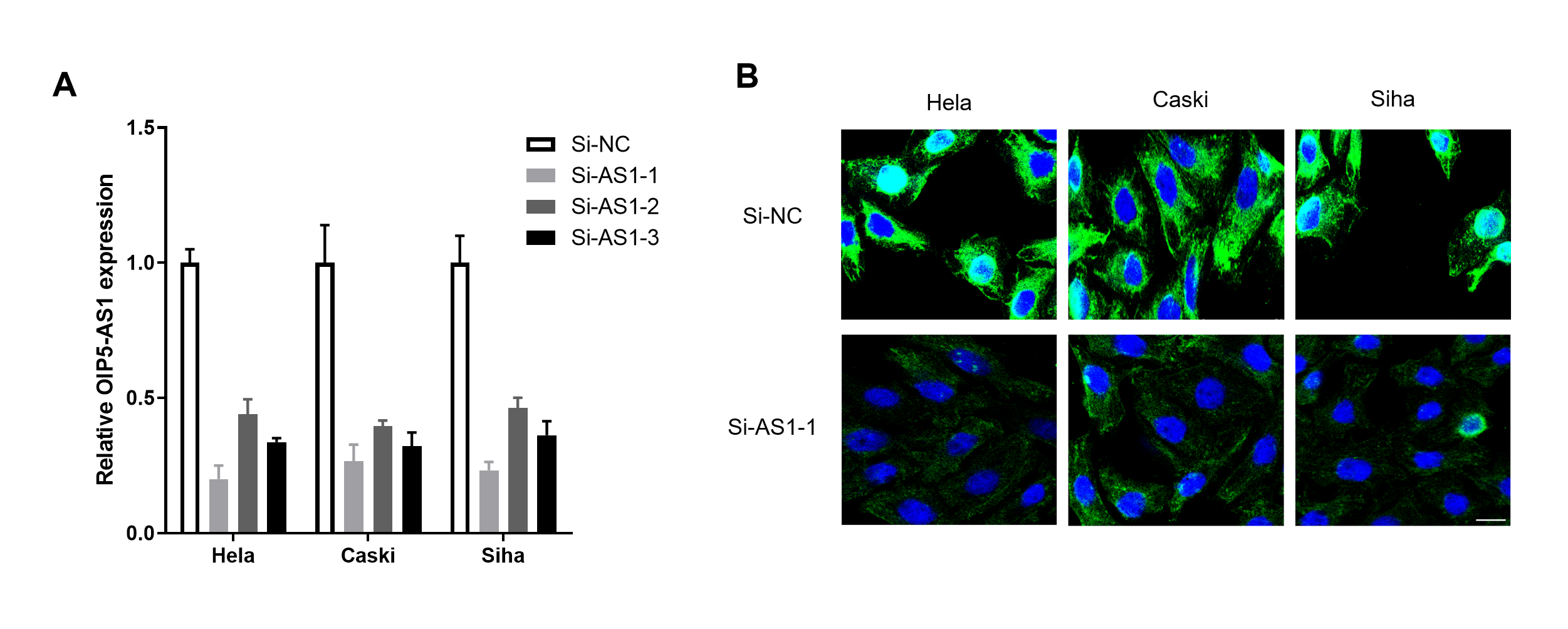

Supplement: Supplementary Figure 1 — Validation of Hela, Caski, and Siha cell line knocking down OIP5-AS1 using RT-qPCR (A) and RT-qPCR (B). [file Image_1.TIF]

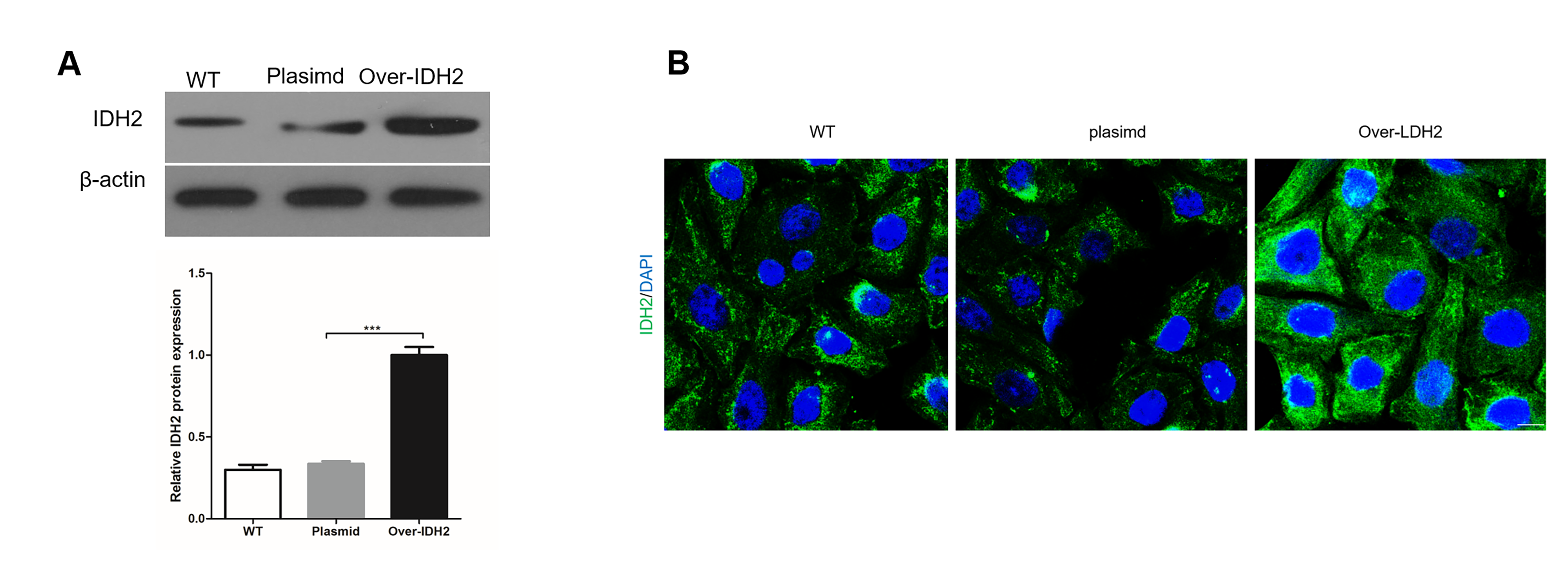

Supplement: Supplementary Figure 2 — Validation of Hela cell line overexpressing IDH2 using western blot (A) and cellular immunofluorescence (B). [file Image_2.TIF]
